# Structural elucidation of the O-antigen polysaccharide from *Escherichia coli* O125ac and biosynthetic aspects thereof

**DOI:** 10.1093/glycob/cwac061

**Published:** 2022-09-10

**Authors:** Axel Furevi, Klas I Udekwu, Göran Widmalm

**Affiliations:** Department of Organic Chemistry, Arrhenius Laboratory, Stockholm University, SE-106 91 Stockholm, Sweden; Department of Aquatic Sciences and Assessment, Swedish University of Agriculture, P.O. Box 7050, SE-750 07 Uppsala, Sweden; Department of Organic Chemistry, Arrhenius Laboratory, Stockholm University, SE-106 91 Stockholm, Sweden

**Keywords:** CarbBuilder, CASPER, lipopolysaccharide, NMR spectroscopy

## Abstract

Enteropathogenic *Escherichia coli* O125, the cause of infectious diarrheal disease, is comprised of two serogroups, viz., O125ab and O125ac, which display the aggregative adherence pattern with epithelial cells. Herein, the structure of the O-antigen polysaccharide from *E. coli* O125ac:H6 has been elucidated. Sugar analysis revealed the presence of fucose, mannose, galactose and *N*-acetyl-galactosamine as major components. Unassigned ^1^H and ^13^C NMR data from one- and two-dimensional NMR experiments of the O125ac O-antigen in conjunction with sugar components were used as input to the CASPER program, which can determine polysaccharide structure in a fully automated way, and resulted in the following branched pentasaccharide structure of the repeating unit: →4)[β-d-Gal*p*-(1 → 3)]-β-d-Gal*p*NAc-(1 → 2)-α-d-Man*p*-(1 → 3)-α-l-Fuc*p*-(1 → 3)-α-d-Gal*p*NAc-(1→, where the side chain is denoted by square brackets. The proposed O-antigen structure was confirmed by ^1^H and ^13^C NMR chemical shift assignments and determination of interresidue connectivities. Based on this structure, that of the O125ab O-antigen, which consists of hexasaccharide repeating units with an additional glucosyl group, was possible to establish in a semi-automated fashion by CASPER. The putative existence of *gnu* and *gne* in the gene clusters of the O125 serogroups is manifested by *N*-acetyl-d-galactosamine residues as the initial sugar residue of the biological repeating unit as well as within the repeating unit. The close similarity between O-antigen structures is consistent with the presence of two subgroups in the *E. coli* O125 serogroup.

## Introduction

*Escherichia coli* is the most abundant of the facultatively anaerobic microbes in the gastrointestinal tract of mammals, colonizing human infants within hours of birth (Kaper et al. 2004). Both commensal and pathogenic strains of this bacterium have been identified in vertebrate hosts, birds, cheeses and several other environments (van Elsas et al. 2011), indicative of high ecological adaptability (Bergthorsson and Ochman 1998). While incompletely understood, the primary ecological niche of *E. coli* in mammals is the mucus layer of the gastrointestinal tract, ostensibly due to its proficient utilization of gluconate as a primary nutrient source.

Pathogenic *E. coli* strains are divided into “pathotypes” distinguishable by serotyping, analysis of lipopolysaccharide (O) and flagellar (H) antigens, and several of these are causative agents of diarrhea, cystitis, pyelonephritis and meningitis (Stenutz et al. 2006; Croxen et al. 2013; Liu et al. 2020). Of the six identified enteric pathotypes, enteropathogenic *E. coli* (EPEC) are associated with severe infections of the gastrointestinal tract in humans. The O125 serogroup, originally identified during a diarrheal outbreak in London in 1952 (Taylor and Charter 1952; Mc Naught 1956) has since been isolated from diarrheal patients worldwide (Croxen et al. 2013) and is recognized as a significant pathogenic EPEC serogroup by the World Health Organization. Recent studies have shown that *E. coli* O125 is comprised of two subgroups determined by O antigen variation, viz., *E. coli* O125ab and *E. coli* O125ac. (Freitas Do Valle et al. 1997) While the original O125 identified was of O125ab type, the O125ac has increasingly been isolated from diarrheal patients worldwide though it is as yet unclear how virulence determinants interplay with serotype in pathogenesis. *E. coli* O125ac strains are clinically termed atypical EPEC as they lack the discriminating adherence factor, EAF, yet display aggregative adherence to HeLa cells, in this case attributed to another gene, *eaeA* (Freitas Do Valle et al. 1997; Elias et al. 2002). Elucidation of the structure of the O antigen of this strain is of particular relevance to the understanding of its increasing geographical prevalence.

To this end, we have performed chemical analysis and NMR spectroscopy experiments, in conjunction with automated structure analysis using the computer program CASPER (Furevi et al. 2022), to determine the O-antigen structure of *E. coli* O125ac in an efficient way. Furthermore, chemical analysis and acquired NMR spectra of the O125ab polysaccharide were utilized in the analysis of the O-antigen structure, relying on information from the structural elucidation of the *E. coli* O125ac O-antigen.

**Fig. 1:**
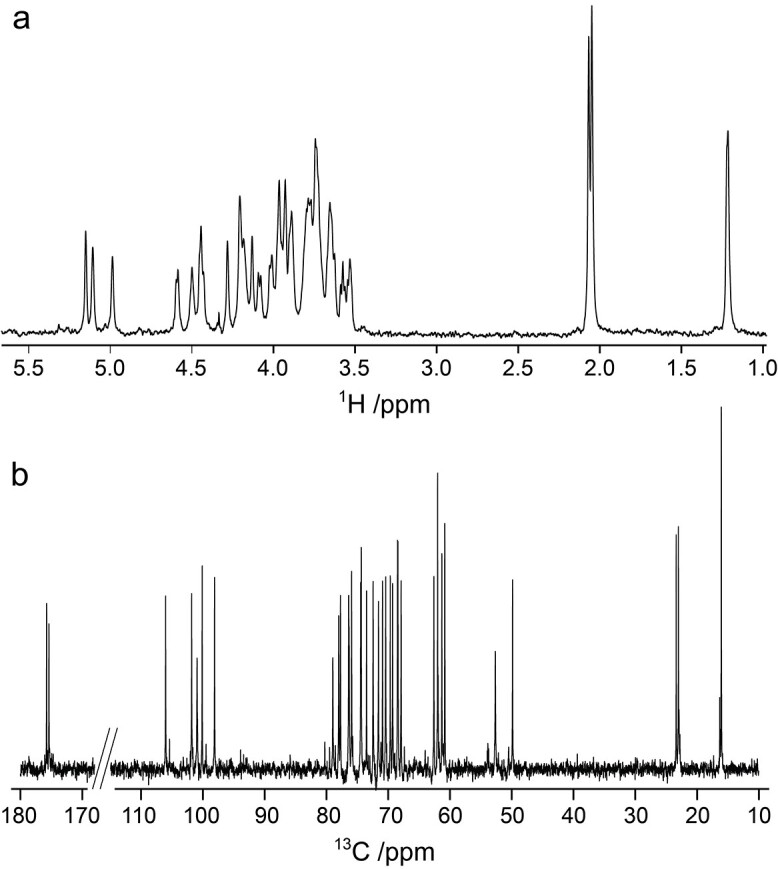
(a) Diffusion-edited ^1^H NMR spectrum and (b) ^13^C NMR spectrum of the *E. coli* O125ac O-antigen polysaccharide.

## Results and discussion

The two *E. coli* strains O125ab and O125ac were cultivated and extracted as previously described (Furevi et al. 2020) in order to obtain lipopolysaccharide (LPS) material. Initial ^1^H NMR spectra of the two LPS did not show any presence of *O*-acetyl groups with distinct resonances at ~2.1 ppm. A mild acidic delipidation, followed by dialysis of both strains, was performed to obtain the polysaccharide materials (PS). Sugar analysis of the *E. coli* O125ab and O125ac PS revealed fucose, mannose, glucose, galactose, galactosamine (Supplementary Fig. S1), but with a significantly lower amount of glucose in O125ac. All the identified sugar-residues had *m*/*z* fragmentation patterns corresponding to their alditol acetates counterparts (Jansson et al. 1976). The structure of the repeating unit (RU) of *E. coli* O125ab has been determined (Kjellberg et al. 1996) and consists of a hexasaccharide RU in which the fucose residue has the l absolute configuration and the remaining sugars have the d absolute configuration. As the gene sequences of the two strains, between *galF* and *gnd*, are closely similar or identical (DebRoy et al. 2016), we concluded that the sugar residues have the same absolute configurations in the PS of the two strains.

The ^1^H NMR spectrum of O125ac PS (Fig. 1a) showed resonances at *δ*_H_ 1.22, 2.07 and 2.05 each corresponding to three protons consistent with the methyl group of C6 in fucose and *N*-acetylation of the two galactosamine residues, respectively. In the spectral region 4.4–5.2 ppm six ^1^H resonances were present, but the ^13^C NMR spectrum (Fig. 1b) showed only five major peaks in the region 98–106 ppm where resonances from anomeric carbon nuclei reside; in total 34 resonances were observed including, inter alia, two from carbonyl carbons at *δ*_C_ 175.37 and 175.76. Taken together these results confirm a pentasaccharide RU containing two *N*-acetyl-d-galactosamine sugars.

**Fig. 2:**
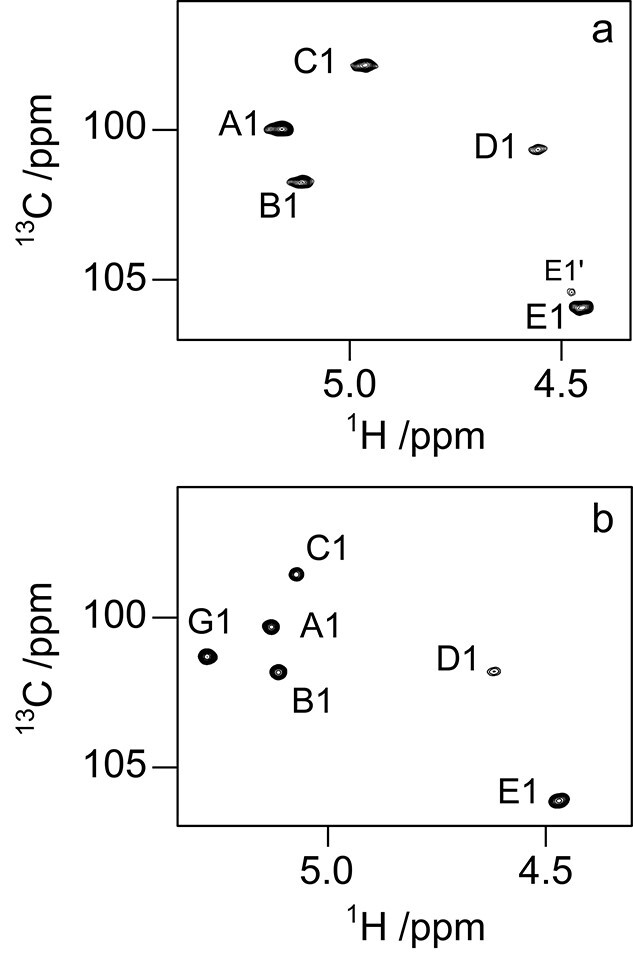
Selected region of anomeric resonances in ^1^H,^13^C-HSQC NMR spectra from *E. coli* O125ac (a) and O125ab (b) O-antigen polysaccharides. Sugar residues are annotated **A** – **E** (**E’**) in order of decreasing ^1^H NMR chemical shifts, except for the additional glucose residue in O125ab that is annotated as **G**.

The ^1^H,^13^C-HSQC NMR spectrum of the anomeric region of the O125ac PS (Fig. 2a) revealed five sugar residues that were annotated **A**–**E** based on descending ^1^H NMR chemical shifts. Moreover, the 2D NMR spectrum also showed two correlations at *δ*_H_/*δ*_C_ 4.44/49.85 and 4.20/52.63 that are characteristic for amino-sugars, as well as a cross-peak at *δ*_H_/*δ*_C_ 1.22/16.05 confirming the presence of the 6-deoxy sugar fucose in the repeating unit. As expected, the anomeric region of O125ab PS displayed correlations for six sugar residues in the region for anomeric resonances (Fig. 2b) and these were herein annotated **A**–**E** and **G** (for glucose); the ^1^H and ^13^C NMR chemical shifts obtained herein correlated well with the previously published data (Kjellberg et al. 1996).

**Fig. 3:**
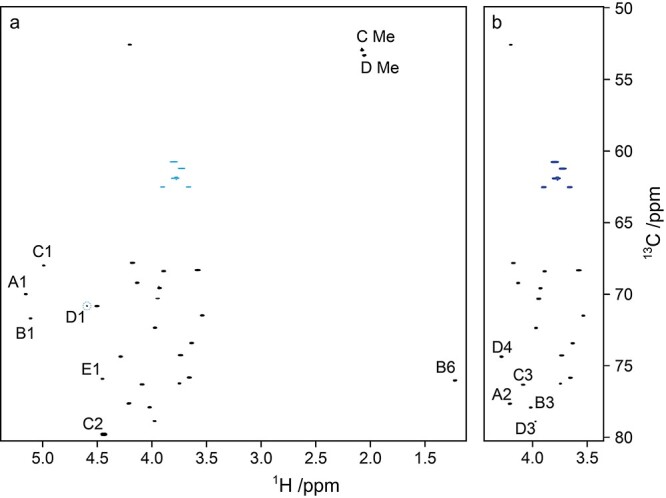
Selected regions of the *E. coli* O125ac O-antigen polysaccharide: (a) an aliased and uniformly sampled ^1^H,^13^C-HSQC NMR spectrum covering 30 ppm in the *F*_1_ dimension, with aliased resonances annotated; (b) a non-uniformly sampled ^1^H,^13^C-HSQC NMR spectrum covering 100 ppm in the *F*_1_ dimension with a sampling coverage of 25 percent, where substitution positions in sugar residues are annotated.

In structural determination of polysaccharides, the spectral resolution in 2D NMR spectra is essential in order to resolve and identify all or a sufficient number of cross peaks for spectral assignment as well as for input to computerized analysis methods. Correlations of ^1^H and ^13^C resonances are most commonly performed by ^1^H,^13^C-HSQC NMR experiments due to higher sensitivity of ^1^H nuclei although the ^13^C-detected ^13^C,^1^H-HETCOR experiment offers a good alternative, sensitivity permitting. To investigate ease of use and quality of ^1^H,^13^C-HSQC NMR spectra these were recorded using on the one hand aliasing in the *F*_1_ dimension and on the other by employing non-uniform sampling in the indirect dimension. The initial ^1^H,^13^C-HSQC NMR experiments of the O125ab and O125ac polysaccharides were used to map the cross-peaks of the resulting spectra and were carried out at a ^1^H frequency of 500 MHz covering 100 ppm with 256 data points and 140 ppm with 512 data points in the *F*_1_ dimension, respectively. To increase resolution in the indirect dimension, two approaches were carried out at 700 MHz, viz., first by recording the ^1^H,^13^C-HSQC spectrum of the O125ac PS using a narrower ^13^C spectral region of 30 ppm covered by 1k data points resulting in aliasing (Fig. 3a), since echo-antiecho gradient selection was used (Jeannerat 2011). In the second case, the spectrum (Fig. 3b) was recorded over 100 ppm using the same number of data points in the *F*_1_ dimension, but with non-uniform sampling (NUS) (Robson et al. 2019) set to 25 percent using a ^13^C *T*_2_ value of 0.1 s, based on transverse relaxation times for polysaccharides at elevated temperature and the same magnetic field strength of 16.4 T (Fontana et al. 2014). In both cases, the resulting acquisition time was ~0.1 s in the indirect dimension and the resolution was similar in the two data sets. Interpretation of the spectrum containing aliased cross-peaks requires calculation of the chemical shift from which the ^13^C resonance originates according to *δ*_0_ = *δ*_a_ ± *n* × SW_ppm_ where *δ*_0_ is the true chemical shift, *δ*_a_ is the apparent chemical shift and *n* is the unknown aliasing order (*n* = 1 herein) and SW_ppm_ is the spectral width in ppm (Jeannerat 2011). In the case of the NUS data, reconstruction of the full 2D data matrix is required prior to Fourier transformation (Mobli et al. 2012). In both spectra, all anticipated correlations can be identified (Fig. 3). The peak shape of the ^1^H,^13^C-correlations was not perturbed in the spectrum for which uniform sampling was employed and where aliasing took place whereas it was slightly perturbed in the spectrum where NUS was utilized, though the spectral appearance is straightforward in the latter case. The well-resolved ^1^H,^13^C-HSQC NMR spectrum of O125ac PS facilitated peak-picking to obtain the ^1^H,^13^C-correlations for subsequent use in determination of the structure of the RU of the polysaccharide.

**Scheme 1:**
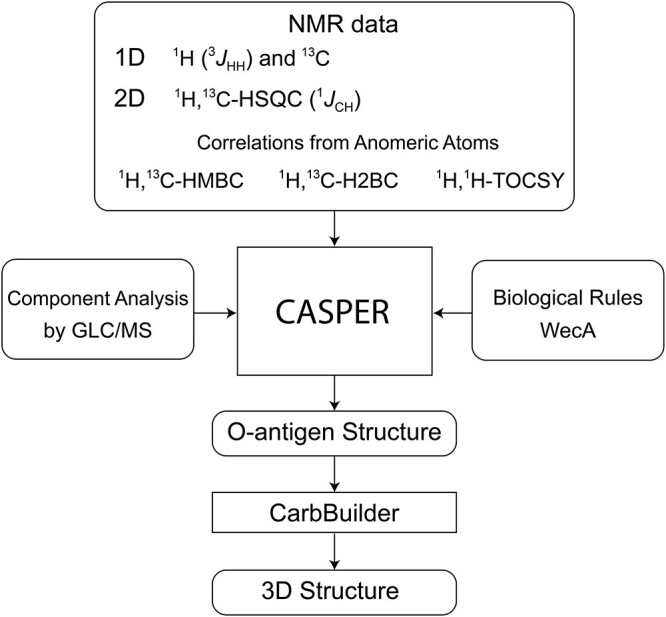
Flowchart of the general workflow used for elucidation of polysaccharide structure using CASPER followed by 3D model building employing CarbBuilder.

Determination of the polysaccharide structure using sugar components and unassigned NMR data can be carried out by the computer program CASPER (Jansson et al. 2006; Lundborg and Widmalm 2011) as shown in Scheme 1. For the *E. coli* O125ac PS, the magnitude of ^3^*J*_H1,H2_ of anomeric protons in the 1D ^1^H NMR spectrum (1 small, 2 medium and 2 large) and chemical shifts from the ^13^C NMR spectrum were entered. From 2D NMR data correlations observed in ^1^H,^1^H-TOCSY, ^1^H,^13^C-HSQC, ^1^H,^13^C-H2BC, ^1^H,^13^C-HMBC spectra were used as input to the program (Supporting information) as well as ^1^*J*_C1,H1_ of anomeric nuclei (2 small and 3 large) from an *F*_2_-coupled ^1^H,^13^C-HSQC spectrum. Additional input that can be provided is information on sugar components, and if the Wzx/Wzy biosynthetic pathway can be anticipated (Ståhle and Widmalm 2019). The latter utilizes WecA resulting in either an *N*-acetyl-d-glucosamine or an *N*-acetyl-d-galactosamine at the reducing end of its biological repeating unit (BRU). This “biological rule” related to WecA should pertain to the *E. coli* O125ac O-antigen possessing four different sugar residues in its RU as seen from the sugar analysis. The NMR data from the above described experiments, the “activated” WecA rule in conjunction with l-Fuc, d-Man, d-Gal and two d-GalNAc as monosaccharide residues, along with all possible linkages available were used as input to the CASPER program using the module “determine glycan structure”.

**Fig. 4:**
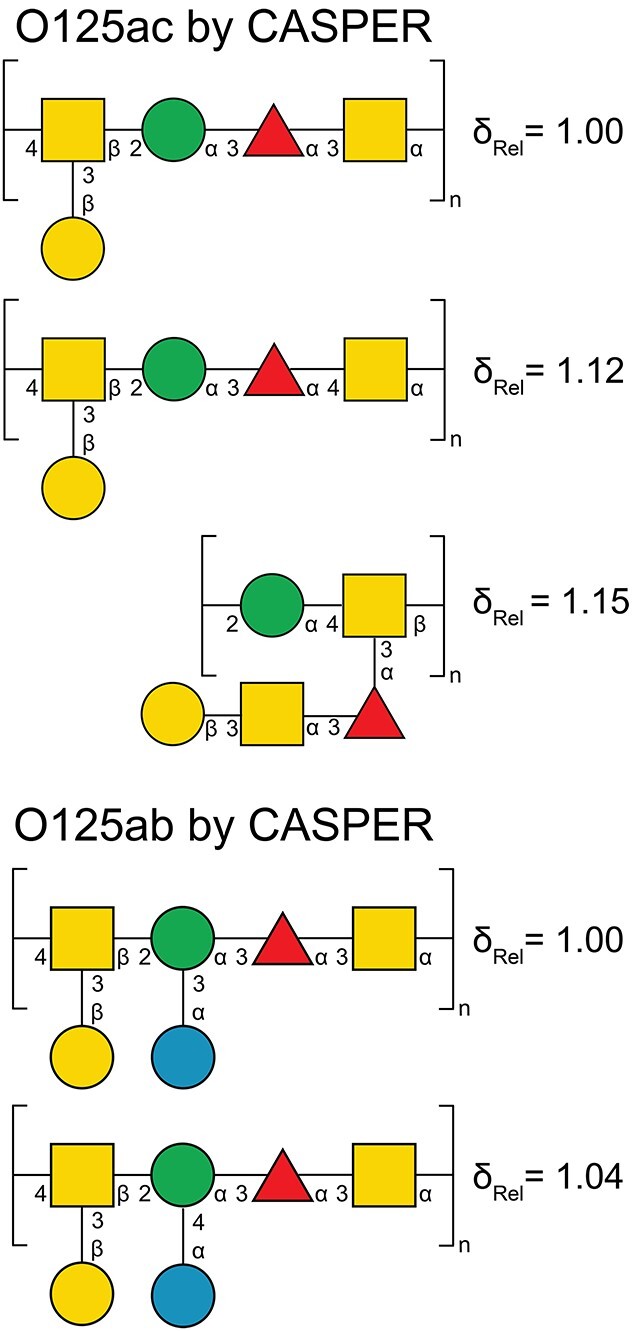
The highest ranked structures using the “determine glycan structure” module in CASPER for the O125ac (top) and the O125ab (bottom) O-antigen polysaccharides. The relative deviations between structures have been normalized by setting the top-ranked structure to unity.

**Fig 5:**
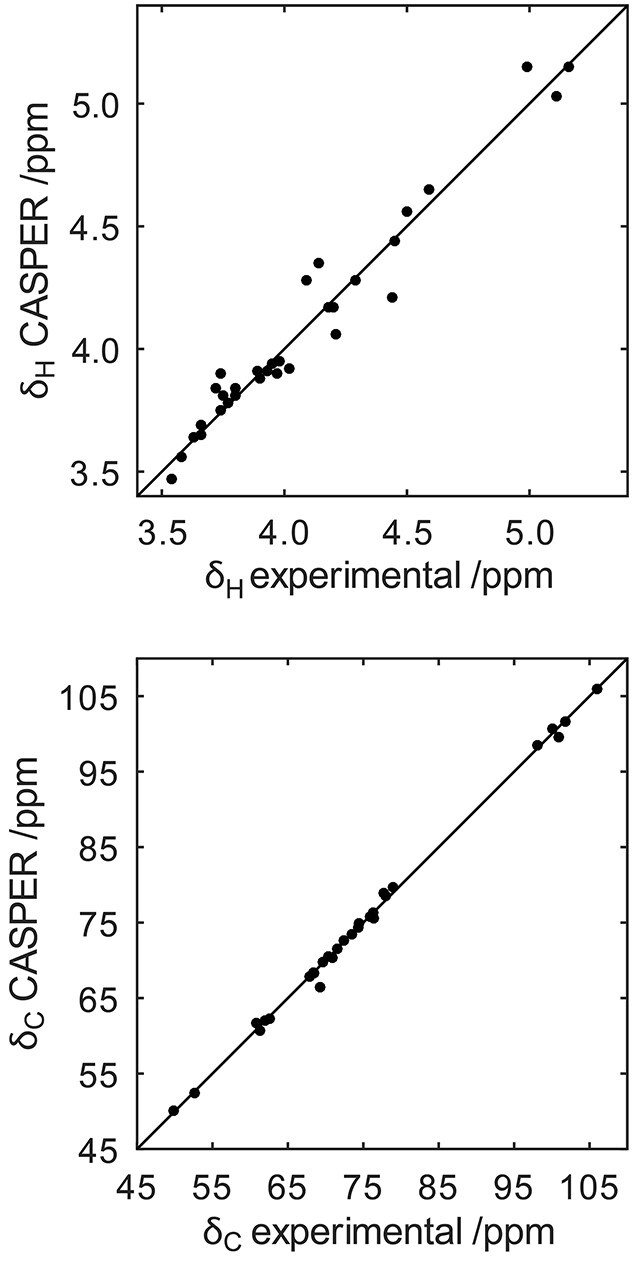
Comparison of ^1^H (top) and ^13^C (bottom) NMR chemical shifts predicted by CASPER versus assigned from NMR experiments for the O125ac O-antigen polysaccharide.

Of the three highest ranked structures for the O125ac PS (Fig. 4) the top-two structures have four sugars in the backbone and one sugar in the side-chain of the RU, i.e. the most commonly observed topology (Liu et al. 2020), and the highest ranked structure contains the same glycosidic linkages as the O125ab PS, except for that of the α-d-Glc residue. The assigned experimental ^1^H and ^13^C NMR chemical shifts (vide infra) correlated quite well with the chemical shifts predicted by CASPER (Fig. 5), although chemical shift deviations were evident, e.g. H2, H3 and H4 of the →3)-α-d-Gal*p*NAc-(1→ residue, which substitutes the →3,4)-β-d-Gal*p*NAc-(1→ residue where branching takes place in the RU. This divergence of ^1^H chemical shifts is presumably due to a lack of model substances in the database for this vicinally disubstituted arrangement containing two *N*-acetyl-d-galactosamine residues, which may lead to a highly congested local structure in the polysaccharide.

Similarly, unassigned NMR input data for the O125ab O-antigen obtained from the herein collected ^1^H,^13^C-HSQC NMR spectrum and from previously described ^1^H,^13^C-HMBC connectivities together with sugar components (Kjellberg et al. 1996), the “activated” WecA rule and information on determined linkage positions and anomeric configurations (as given above) present in the O125ac O-antigen structure were used to elucidate the O125ab O-antigen structure by CASPER, i.e. determining the linkage and anomeric configuration of the additional d-glucosyl residue (Fig. 4). This two-step process relies first on the structure of the O125ac O-antigen and then adding data to obtain the structure of the O125ab O-antigen, akin to the NMR structure determination of *O*-acetyl substitution in the O-antigen of *Salmonella typhimurun* O-factor 5 LPS relying on the O-antigen structure from *S. typhimurun* O-factor 4 (Ståhle and Widmalm 2017). However, it was not possible to fully utilize the outlined approach. Instead, when the sugar residue to which the glucosyl residue should be linked was restricted to the mannosyl residue using “unknown linkage position,” the highest ranked structure predicted by CASPER (Fig. 4) agreed with the previously elucidated O-antigen structure of *E. coli* O125ab (Kjellberg et al. 1996).

Complete assignment of NMR resonances (Table 1) was performed in order to confirm the suggested structure of the O125ac O-antigen polysaccharide, using herein a nomenclature referring to the residue by a capital letter, atom position in the sugar residue by a number, where the number may also correspond to a resonance from a ^1^H or ^13^C nucleus or a ^1^H/^13^C correlated pair. Analysis of ^13^C NMR chemical shifts showed absence of furanosides (Ritchie et al. 1975; (Beier et al. 1980) and that all sugar residues have the pyranoid ring-form (Jansson et al. 1989; Jansson et al. 1991) in the O125ac PS. The anomeric configuration of each sugar residue in the O125ac PS was assigned from the magnitude of ^1^*J*_C1,H1_ (Bundle and Lemieux 1976) observed in an *F*_2_-coupled ^1^H,^13^C-HSQC spectrum. Correlations for the anomeric nuclei corresponding to **A1**, **B1** and **C1** show ^1^*J*_C1,H1_ > 168 Hz, and these sugar residues thus have the α-anomeric configuration, whereas the correlations for **D1** and **E1** have ^1^*J*_C1,H1_ of 165 and 162 Hz, respectively, and consequently these sugar residues have the β-anomeric configuration. The ^1^H NMR chemical shifts of the anomeric resonances of the O125ac PS were resolved and identification of the sugar residues and whether they had a *gluco*/*galacto*- or *manno*-configuration was aided by an array of ^1^H,^1^H-TOCSY spectra using mixing times of 30, 60, 90, 120 and 200 ms.

**Table 1:** ^1^H and ^13^C NMR chemical shifts of the O-antigen from *E. coli* O125ac

	Sugar residue	1	2	3	4	5	6	Me	CO
**A**	→2)-α-d-Man*p*-(1→	5.16	4.21	3.95	3.58	3.74	3.66, 3.90		
		(−0.02)	(0.27)	(0.09)	(−0.10)	(−0.08)			
		100.08 [172]	77.71	70.37	68.39	74.33	62.58		
		(5.14)	(6.02)	(−0.88)	(0.45)	(1.00)			
**B**	→3)-α-l-Fuc*p*-(1→	5.11	3.89	4.02	3.97	4.18	1.22		
		(−0.09)	(0.12)	(0.16)	(0.16)	(−0.02)			
		101.79 [172]	68.46	77.98	72.42	67.88	16.05		
		(8.67)	(−0.63)	(7.68)	(−0.38)	(0.78)	(−0.28)		
**C**	→3)-α-d-Gal*p*NAc-(1→	4.99	4.44	4.09	4.14	4.50	~3.80	2.07	
		(−0.29)	(0.25)	(0.14)	(0.09)	(0.37)			
		98.09 [174]	49.85	76.38	69.27	70.90	60.82	23.01	175.37
		(6.14)	(−1.31)	(7.98)	(−0.27)	(−0.46)	(−1.29)		
**D**	→3,4)-β-d-Gal*p*NAc-(1→	4.59^a^	4.20	3.98	4.29	3.75	3.72, 3.74	2.05	
		(−0.09)	(0.30)	(0.21)	(0.31)	(0.03)			
		100.91 [165]	52.63	78.94	74.43	76.31	61.29	23.36	175.75
		(4.62)	(−2.17)	(6.93)	(5.60)	(0.33)	(−0.60)		
**E**	β-d-Gal*p*-(1→	4.45	3.54	3.63	3.93	3.66	3.77, 3.80		
		(−0.08)	(0.09)	(0.04)	(0.04)	(0.01)			
		106.00 [162]	71.55	73.48	69.64	75.90	61.97		
		(8.63)	(−1.41)	(−0.30)	(−0.05)	(−0.03)	(0.13)		

*J*_C1,H1_ are given in hertz in square brackets. ^1^H and ^13^C NMR chemical shift differences between experimental data and corresponding monosaccharides (Jansson et al. 1989) are displayed in parentheses.

a *J*_H1,H2_ = 8.3 Hz.

In ^1^H,^1^H-TOCSY spectra **A1** showed only one cross-peak to *δ*_H_ 4.21 at mixing times 30–120 ms, while at 200 ms, five correlations were observed, which indicated a spin-system from a sugar having the *manno*-configuration. From the resonance frequencies for **B1** and **C1** one cross-peak was detected at *δ*_H_ 3.89 and 4.44, respectively, for the shortest mixing time of 30 ms; longer mixing times of 60–200 ms revealed three correlations (H2–H4 from each sugar residue) indicating spin-systems from residues having the *galacto*-configuration. Arising from **D1** and **E1** two correlations were observed for each sugar residue when the shortest mixing time of 30 ms was used, viz., to *δ*_H_ 4.20 and 3.98 and to δ_H_ 3.54 and 3.63, respectively; in each of the spin systems, a third cross-peak was observed to *δ*_H_ 4.29 (**D4**) and to *δ*_H_ 3.93 (**E4**) when mixing times in the range 60–200 ms were used. The poorer transfer of magnetization in **B1**/**C1** compared to **D1**/**E1** is consistent with the former having the α-anomeric configuration and the latter, the β-anomeric configuration, in complete agreement with the results from ^1^*J*_C1,H1_ coupling constants of the sugar residues. This analysis also confirms the lack of a sugar residue having the *gluco*-configuration in the RU, and consequently the O125ac PS is devoid of a glucosyl residue in its O-antigen.

The spin-systems were thereafter fully assigned using a combination of ^1^H,^13^C-HSQC, ^1^H,^13^C-H2BC, ^1^H,^13^C-HMBC and ^1^H,^13^C-HSQC-TOCSY experiments; the two experiments with TOCSY transfer employed mixing times of 20 and 120 ms, respectively. Starting from **A1** correlations were observed to *δ*_H_/*δ*_C_ 4.21/77.71 (**A2**) via cross-peaks in both ^1^H,^13^C-H2BC and ^1^H,^13^C-HSQC-TOCSY spectra and further from **A2** cross-peaks throughout the spin-system were observed due to large ^3^*J*_H,H_ coupling constants from H2 and onward in mannosyl residues; combined with information form ^1^H,^13^C-H2BC spectra resonances from **A3**–**A6** could thus be fully assigned.

The spin-system of the fucosyl residue was tied together starting from the methyl group resonance of the 6-deoxy sugar resonating at *δ*_H_/*δ*_C_ 1.22/16.05 (**B6**) with subsequent correlations observed in the ^1^H,^13^C-H2BC and ^1^H,^13^C-HSQC-TOCSY spectra to *δ*_H_/*δ*_C_ 4.18/67.88 (**B5**) as well as a ^1^H,^13^C-HMBC correlation due to ^3^*J*_H6,C4_ observed at *δ*_C_ 72.42 corresponding to **B4**. From anomeric positions **C1** and **D1** both display cross-peaks in the ^1^H,^13^C-H2BC spectrum to *δ*_H_/*δ*_C_ 4.44/49.85 (**C2**) and *δ*_H_/*δ*_C_ 4.20/52.63 (**D2**), respectively, the ^13^C NMR chemical shifts of which are characteristic of 2-amino-2-deoxy-sugars (Jansson et al. 1989). In the ^1^H,^13^C-BS-CT-HMBC spectrum correlations were observed between the resonance at *δ*_C_ 175.37 and **C2**, and from *δ*_C_ 175.75 to **D2**. Thus, residues **C** and **D** can be assigned to α-d-GalNAc and β-d-GalNAc, respectively.

**Fig. 6:**
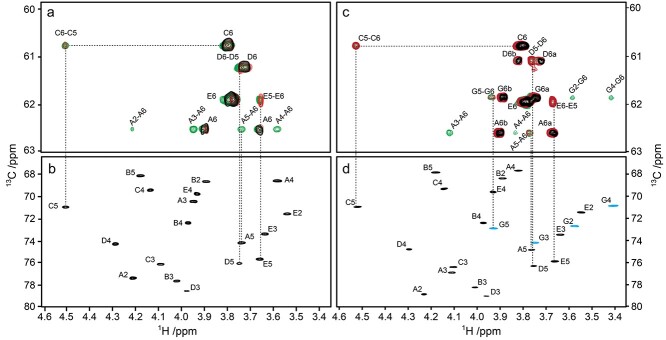
Selected regions with (a) multiplicity-edited ^1^H,^13^C-HSQC NMR spectrum of the O125ac O-antigen polysaccharide (correlations in black) overlaid with ^1^H,^13^C-HSQC-TOCSY spectra with mixing times of 20 (correlations in red) and 120 ms (correlations in green); (b) multiplicity-edited ^1^H,^13^C-HSQC NMR spectrum of O125ac PS with assigned correlations; (c) ^1^H,^13^C-HSQC NMR spectra of O125ab PS (correlations in black) overlaid with ^1^H,^13^C-HSQC-TOCSY spectra with mixing times of 20 ms (red) and 120 ms (green); (d) multiplicity-edited ^1^H,^13^C-HSQC NMR spectrum of O125ab PS with assigned correlations, where those from the glucose residue, **G2**-**G5**, are highlighted in blue. Note the different expansions in the ^13^C dimension of the of the spectral region related to hydroxymethyl groups (top) and cross-peaks related to ring-carbon resonances (bottom).

The high resolution in the ^13^C dimension of the ^1^H,^13^C-HSQC and ^1^H,^13^C-HSQC-TOCSY (20 and 120 ms mixing times) spectra facilitated chemical shift assignments for the hydroxymethyl groups of residues **A**, **C**, **D** and **E** in both polysaccharides as well as the one in residue **G** of the O125ab PS to be fully assigned (Fig. 6). Interestingly, the proton chemical shifts of **D6a** and **D6b** were closely similar, *δ*_H_ 3.72 and *δ*_H_ 3.74, respectively, in the O125ac PS but showed a larger difference for the O125ab PS, viz., **D6a** and **D6b** having *δ*_H_ 3.72 and *δ*_H_ 3.82, respectively, presumably due to a change in the rotameric distribution of the ω (O5-C5-C6-O6) torsion angle of the branched →3,4)-β-d-Gal*p*NAc-(1 → when the additional glucosyl group is present and linked to the mannosyl residue in the backbone of the polysaccharide. The NMR chemical shift assignments of hydroxymethyl groups of residues in the O125ab PS carried out herein complement the previous study (Kjellberg et al. 1996) and are given by *δ*_H6a_/*δ*_H6b_/*δ*_C6_ 3.66/3.90/62.60 (**A**), 3.79/3.81/60.80 (**C**), 3.72/3.82/61.09 (**D**), 3.77/3.79/61.98 (**E**) and 3.75/3.88/61.87 (**G**).

**Fig. 7:**
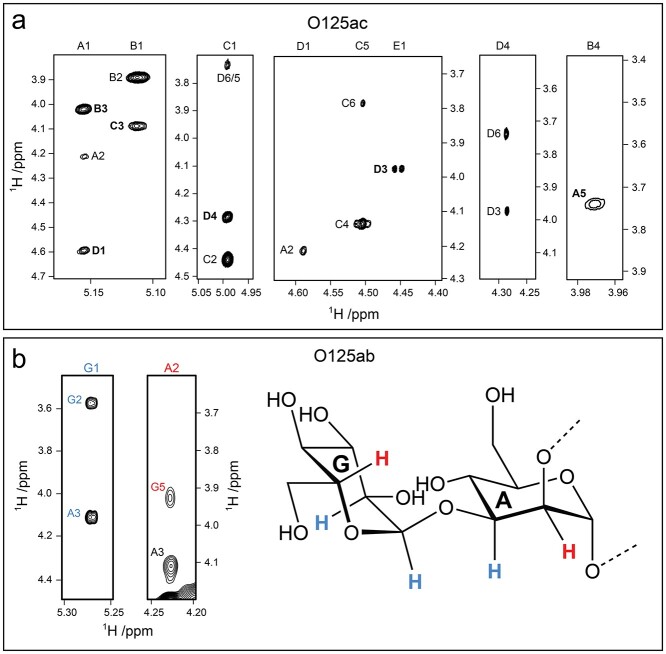
(a) Selected regions of a ^1^H,^1^H-NOESY NMR spectrum at 700 MHz with a 60 ms mixing time for the O125ac O-antigen polysaccharide. Trans-glycosidic or inter-residue correlations are annotated in bold; (b) selected regions of a ^1^H,^1^H-NOESY NMR spectrum at 700 MHz with a 60 ms mixing time for the O125ab polysaccharide along with a schematic representation of residues **G** and **A**. Observed correlations between atom pairs **G1**-**G2** and **G1**-**A3** are highlighted in blue and the one for **A2**-**G5** is marked in red.

The linkage positions of the O125ac PS suggested by CASPER were confirmed experimentally by correlations observed in ^1^H,^13^C-HMBC and ^1^H,^1^H-NOESY spectra (Table 2, Figs 3b and 7a). Significant downfield ^13^C NMR chemical shift displacements in the range 5.6–8.0 ppm were observed for the linkage positions compared to their monosaccharide counterparts (Söderman et al. 1998), which further supported the position(s) of substitution in each sugar residue of the polysaccharide (Table 1).

**Table 2:** Inter-residue correlations observed in ^1^H,^1^H-NOESY and ^1^H,^13^C-HMBC NMR spectra for the *E. coli* O125ac O-antigen polysaccharide

	Sugar residue		NOE	HMBC^a^
		Atom	Correlations to	
**A**	→2)-α-d-Man*p*-(1→	H1,**A**	H3,**B**; H1,**D**	C3,**B**
**B**	→3)-α-l-Fuc*p*-(1→	H1,**B**	H3,**C**	C3,**C**
**C**	→3)-α-d-Gal*p*NAc-(1→	H1,**C**	H4,**D**; H6/H5,**D**	C4,**D**
**D**	→3,4)-β-d-Gal*p*NAc-(1→	H1,**D**	H2,**A** (H2,**D**)	C2,**A**
**E**	β-d-Gal*p*-(1→	H1,**E**	H3,**D**	C3,**D**

a Correlations were also observed for C1,**C** to H4,**D** and C1,**D** to H2,**A** (H2,**D**).

In addition, the ^1^H,^1^H-NOESY spectra of both O125ac (Fig. 7a) and O125ab showed a strong correlation between **A5** and **B4**, which indicated an *exo*-anomeric conformation at the glycosidic linkage for the structural element →2)-α-d-Man*p*-(1 → 3)-α-l-Fuc*p*-(1 → (**A**-**B**). In a polysaccharide model of the O125ac PS generated by CarbBuilder (Kuttel et al. 2016) with torsion angles *ϕ*_H_ = −40° and *ψ*_H_ = 0° for the **A**-**B** structural element had a distance of <3 Å between **A5** and **B4** consistent with the observation of a conspicuous cross-peak in the ^1^H,^1^H-NOESY spectrum (Fig. 7a, rightmost panel).

Analysis of the ^1^H,^1^H-NOESY spectrum of the O125ab PS whose RU contains an additional glucosyl residue (**G**) showed, besides from the expected trans-glycosidic linkage correlation, **G1** to **A3**, an inter-residual correlation between **A2** and **G5** (Fig. 7b). Integration of cross-peak volumes in the ^1^H,^1^H-NOESY spectrum (700 MHz and mixing times of 60 and 150 ms) showed that **G1**-**A3 < G1**-**G2 < G5**-**A2**. Based on molecular model-derived proton-proton distances the structural element α-d-Glc*p*-(1 → 3)-α-d-Man*p*-(1→ displays a slightly different conformational preference compared to the similar structural element α-d-Gal*p*-(1 → 3)-β-d-Man*p*-(1→ in the *E. coli* O188 O-antigen (Furevi et al. 2020) for which the corresponding NOEs were closely similar and the corresponding proton-proton pairs are approximately equidistant.

In the ^1^H,^13^C-HSQC NMR spectrum of the O125ac PS a cross-peak of low intensity was observed close to the cross-peak from the H1/C1 nuclei of side-chain galactose group (**E**), i.e. it was present at *δ*_H_/*δ*_C_ 4.47/105.50 presumed to originate from a similar sugar residue denoted by **E1’**. The NMR chemical shifts of the low intensity cross-peak are in excellent agreement with predictions made by CASPER for the β-d-Gal*p*-(1 → 3)-β-d-Gal*p*NAc disaccharide structural element, i.e. *δ*_H_/*δ*_C_ 4.48/105.50, thereby lending support to that this H1/C1 correlation in the spectrum arises from the terminal sugar residue in the O-antigen, consistent with a d-Gal*p*NAc residue at the reducing end of the biological repeating unit (BRU). Furthermore, the number of RUs of the O-antigen polysaccharide was estimated by integration of the ^13^C resonances from the anomeric carbons of residues **E1** and **E1’** and was found to be ~15.

In the gene clusters of the O125 serogroups (Iguchi et al. 2015; DebRoy et al. 2016; Liu et al. 2020), the presence of *manBC* is consistent with the mannosyl residue in the polysaccharides. Furthermore, in the Wzx/Wzy biosynthetic pathway of O-antigen polysaccharides (Ståhle and Widmalm 2019), Gnu is responsible for converting Und-*PP*-GlcNAc to Und-*PP*-GalNAc when GalNAc is the initial sugar in the BRU and Gne isomerizes UDP-d-GlcNAc to UDP-d-GalNAc (Rush et al. 2010; Cunneen et al. 2013) The presence of two glucose 4-epimerases in the gene cluster of the O125 serogroup (Iguchi et al. 2015), presumably *gnu* and *gne*, is fully consistent with products thereof participating in the biosynthesis of an oligosaccharyl-*PP*-Und entity, which is then flipped into the periplasmic space and polymerized by the Wzy-Wzz complex (Ståhle and Widmalm 2019; Wiseman et al. 2021) prior to translocation and insertion into outer leaflet of the outer membrane by the Lpt pathway (Li et al. 2019; Owens et al. 2019). Furthermore, there are four putative GTs encoded between *galF* and *gnd* in the gene cluster of O125 (Iguchi et al. 2015), viz., *wetD*, *wetE*, *wdaF* and *wdaG* (Liu et al. 2020) and an analysis of conserved domains in these genes revealed them as GTs belonging to families, 2, 2, 4 and 2, respectively, where family 2 acts through an inverting mechanism and family 4 operates via a retaining mechanism. From the pentasaccharide RU of the O125ac O-antigen structure the four NDP-donors used by the GTs should be GDP-l-Fuc, GDP-d-Man, UDP-d-GalNAc and UDP-d-Gal in sequence for the nascent oligosaccharyl-*PP*-Und. Furthermore, GDP-l-Fuc has the β-anomeric configuration whereas the other three NDP-donors have the α-anomeric configuration and in the O-antigen structure the sugar residues Fuc, GalNAc and Gal all have an inverted anomeric configuration compared to that of their NDP-donors. Taken together with the retention of the anomeric configuration for the sugar residue Man, we propose that the third GT in the gene sequence uses GDP-d-Man as the donor in the glycosylation reaction. This glycosylation step also corresponds to the second glycosidic linkage formed for the oligosaccharyl-*PP*-Und. Thus, we surmise that the GTs are present in inverse order of their function order; i.e. the gene for the first sugar to be added corresponds to the last GT gene in the cluster and so on (Liu et al. 2020). If this is the case, the functions have been identified of all the GTs making the O125ac oligosaccharyl-*PP*-Und, whose oligosaccharide subsequently is polymerized. In *E. coli*, translation of a given protein within an operon increases with distance from transcription end (transcription distance) (Lim et al. 2011). While operon arrangement favors production of 5′-end proximal genes more so than downstream encoded open reading frames, the “reverse order arrangement” may be significant in controlling the concentration of lipopolysaccharides in the growing cell.

Additionally, we speculate that the glucosyl group in the O125ab O-antigen is added at the periplasmic side of the inner membrane in a three-step process (Mann and Whitfield 2016) whereby a glucosyl entity is transferred from UDP-glucose to the undecaprenyl phosphate by GtrB to yield the Und-*P*-Glc precursor in the cytoplasm. This is followed by the flipping of Und-*P*-Glc into the periplasm by GtrA and subsequent transfer of the glucosyl group by a specific glucosyl transferase (Gtr) to the acceptor molecule. Notably, this sequence is similar to the glucosylation that occurs in modification of e.g. the *Shigella flexneri* serotype 2a O-antigen (Teh et al. 2020), in which the linear RU of the polysaccharide corresponding to serotype Y is converted by GtrII into serotype 2a having α-d-Glc*p*-(1 → 3)-linked to Rha^I^ of the RU, resulting in branched structures within the O-antigen.

**Fig. 8:**
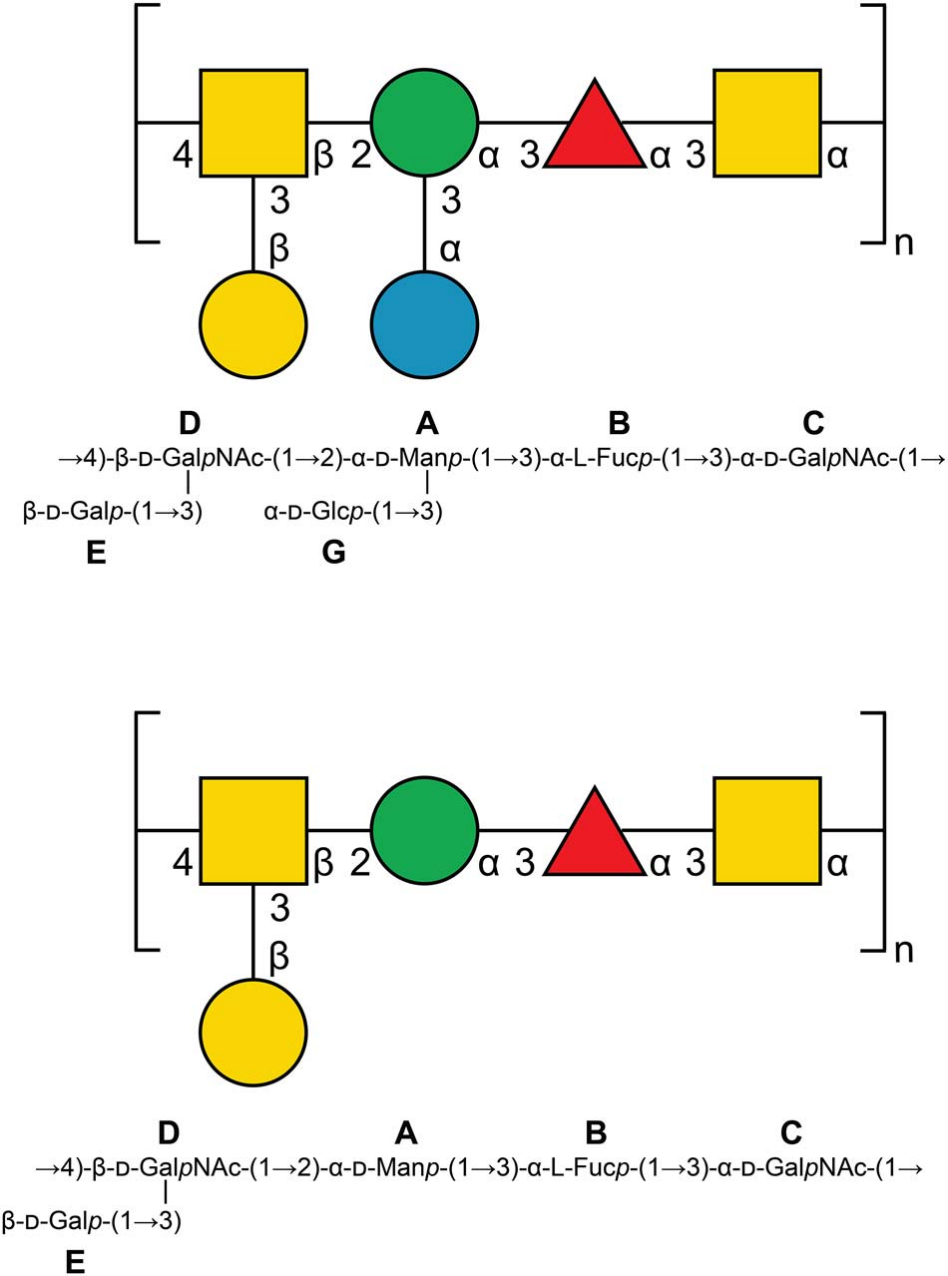
*E. coli* O125ab and O125ac O-antigen repeating units in SNFG-representation (Neelamegham et al. 2019) and in standard nomenclature; sugar residues are labeled by bold capital letters.

**Fig. 9:**
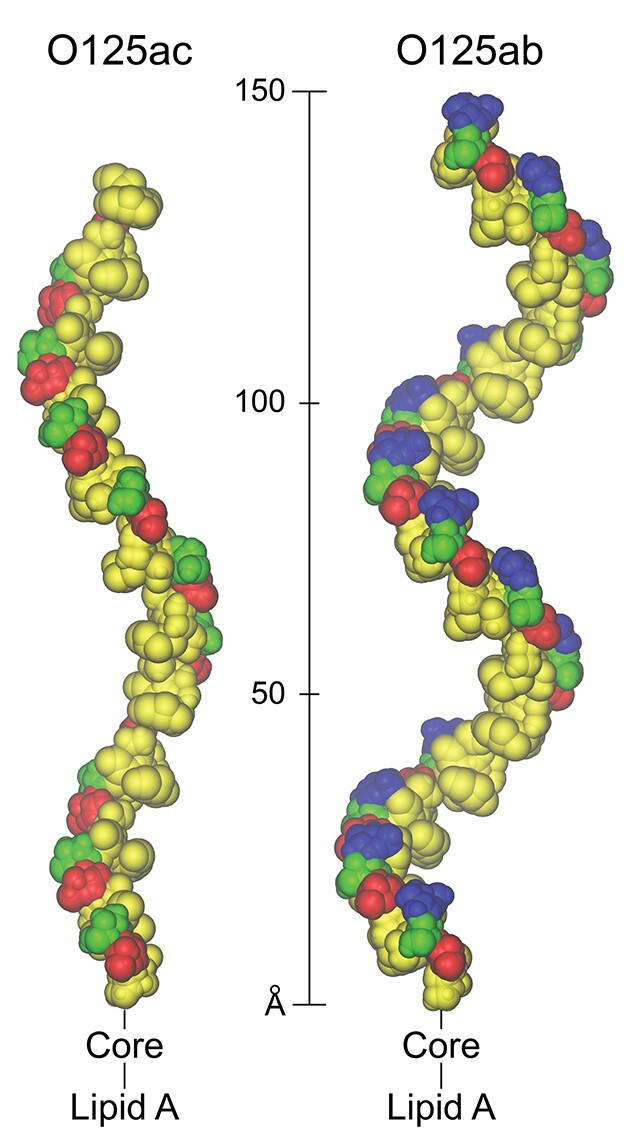
CarbBuilder (Kuttel et al. 2016) models of *E. coli* O125ac and O125ab O-antigen polysaccharides with 11 and 15 repeating units, respectively, visualized by VMD (Humphrey et al. 1996) in a van der Waals’ representation. The sugar residues are colored according to the SNFG-standard (Neelamegham et al. 2019) with Gal/GalNAc residues in yellow, Glc residues in blue, Man residues in green and Fuc residues in red.

The structures of the two O-antigens from *E. coli* O125ab and O125ac (Fig. 8) differ only by the presence or absence of a side-chain glucosyl group, respectively. Molecular models of the polysaccharides generated by CarbBuilder (Fig. 9) reveal possible differences in the backbone conformation of the two polymers as well as the well-exposed epitope presented by the glucosyl group at each RU of the O125ab O-antigen. Not only should it be possible to identify and make a monoclonal antibody that specifically recognizes an epitope that contains the glucosyl group in the RU of the O125ab O-antigen, but results from the modeling suggest that the glucosylation will lead to a shortening of the O-antigen of the LPS molecule, i.e. not extending as far from the outer membrane of the bacterium. This change in polysaccharide extension upon glucosylation is reminiscent of the difference between the O-antigen in *S. flexneri* serotype 5a and that of serotype Y, which is a linear polysaccharide devoid of side-chains. The former extends only half as far as the latter, which has important consequences for the ability of the tip of the filamentous needle of the bacterium to make contact with the host cell membrane in invasion of the gut epithelial cells of mammalian hosts (West et al. 2005). Possibly, a similar mechanism may be at work in *E. coli* O125ab. The degree of extension of the O-antigens of serogroups O125ac and O125ab as part of their LPS at the bacterial membrane surface could be investigated in detail by molecular dynamics simulations (Patel et al. 2020) to shed light on O-antigen modification in general and glucosylation in particular.

## Conclusions

The structure of the O-antigen repeating unit from *E. coli* O125ac was determined using NMR as the main tool. The structure elucidation program, CASPER, successfully predicted its structure to be similar to the O125ab O-antigen, except that the O125ac polysaccharide lacks a glucosyl side-chain residue in its BRU. NMR resonance assignments utilizing multiple 2D NMR experiments confirmed the O-antigen structure. Furthermore, ^1^H,^13^C-HSQC and ^1^H,^13^C-HSQC-TOCSY experiments with high resolution in the *F*_1_ dimension facilitated full assignment of the chemical shifts of the hydroxymethyl groups of hexose residues in both the O125ac and O125ab polysaccharides. Analysis of putative GTs between *galF* and *gnd* in the gene cluster of O125, the proposed GT families and the mechanism by which they act, i.e. inverting or retaining, NDP-donor sugars and their sequence in the gene cluster supports the hypothesis that the genomic arrangement of encoded GTs is inverse to their functional order, i.e. the gene for the last sugar to be added corresponds to the first GT gene in the cluster etc., and consequently we were able to identify the function of the GTs that make the oligosaccharyl-*PP*-Und. Three-dimensional molecular models of the O-antigens highlight the similarity between the structures but also a different epitope arising from the glucosyl group of O125ab, thereby explaining that the *E. coli* O125 serogroup has been subdivided into O125ab and O125ac.

## Materials and methods

### Bacterial strains and polysaccharide preparations

*E. coli* reference strains of O125ac (*E. coli* O125ac:H6) and O125ab (*E. coli* O125ab:H19) were obtained from SSI Diagnostica A/S (Hillerød, Denmark). Bacterial culture, LPS extraction and preparation of lipid-free polysaccharides (PS) were carried out as previously described (Furevi et al. 2020).

### Sugar analysis by GC–MS

Polysaccharides purified by gel-filtration chromatography (PS) from O125ab and O125ac were hydrolyzed with 2 M TFA at 120 °C for 30 min followed by reduction with NaBH_4_ in aq. ammonia (1 M) at ambient temp for 30 min and acetylation at 100 °C for 30 min using Ac_2_O and pyridine (1:1). The resulting mixtures of alditol acetates were analyzed by gas chromatography–mass spectrometry (GC–MS) and separated on an HP-5MS column attached to a Shimadzu GCMS-QP2020 with an electron impact (EI) source. The temperature program used an injection temperature of 250 °C and an initial oven temperature of 60 °C with a hold time of 2 min, followed by ramped heat increase @ 20 °C min^−1^ to 200 °C then 5 °C min^−1^ to 260 °C with a hold time of 4 min at 260 °C. The samples were compared with authentic standard references and their mass fragments. The retention times of the derivatives were compared to those of authentic standards as references and their mass spectra were compared to EI-MS fragmentation patterns (Jansson et al. 1976).

### NMR spectroscopy

The PS samples were deuterium-exchanged by freeze-drying from 99.9 percent D_2_O and examined by NMR spectroscopy as solutions in 99.96 percent D_2_O (8 mg in 0.55 mL) with a trace amount of NaN_3_ prior to 1D ^13^C NMR, 2D ^1^H,^13^C-HMBC, ^1^H,^13^C-H2BC and ^1^H,^13^C-BS-CT-HMBC experiments. Samples of O125ab and O125ac PS purified by gel-permeation chromatography were used for additional NMR experiments (1 mg in 0.55 mL). NMR spectra were recorded at 70 °C using Bruker Avance 500 MHz or Bruker Avance III 700 MHz spectrometers equipped with 5 mm TCI (^1^H/^13^C/^15^N) Z-Gradient (53.0 G cm^−1^) CryoProbes. Chemical shifts are reported in ppm using internal sodium 3-trimethylsilylpropanoate-2,2,3,3-*d*_4_ (TSP, *δ*_H_ 0.00) for ^1^H NMR and external 1,4-dioxane 10 percent in D_2_O (*δ*_C_ 67.40) for ^13^C NMR as references. Chemical shift differences were obtained by comparison to NMR data of the corresponding monosaccharides (Jansson et al. 1989). NMR experiments apt for resonance assignments of carbohydrates (Widmalm 2021) were recorded essentially as previously described (Furevi et al. 2020); specific additional experimental conditions are given below.

The *F*_2_-coupled ^1^H,^13^C-HSQC experiment applied to the O125ac PS was recorded with 1224 × 256 data points in the *F*_2_ and *F*_1_ dimensions, respectively, using an evolution time corresponding to ^1^*J*_CH_ of 145 Hz. Two ^1^H,^1^H-NOESY experiments, with suppression of effects from zero-quantum coherence (Thrippleton and Keeler 2003), were carried out for the O125ab PS at 700 MHz with mixing times of 60 and 150 ms employing 14 k × 256 data points in *F*_2_ and *F*_1_, respectively, an acquisition time of 1.7 s and a relaxation delay of 5 s. Multiplicity-edited ^1^H,^13^C-HSQC-TOCSY experiments with 20 and 120 ms mixing times were recorded with 918 × 1024 data points in the *F*_2_ and *F*_1_ dimensions, respectively, with non-uniform sampling using a sparsity level of 25 percent and an evolution time corresponding to ^1^*J*_CH_ of 145 Hz for both the O125ab PS and the O125ac PS. Multiplicity-edited ^1^H,^13^C-HSQC experiments were acquired for the two polysaccharides at a ^1^H frequency of 500 MHz with 1224 × 512 data points in *F*_2_ and *F*_1_, respectively, covering 140 ppm in the indirect dimension for the O125ac PS and 840 × 256 as well as 938 × 1024 data points in *F*_2_ and *F*_1_, respectively, covering 100 ppm in *F*_1_ for the O125ab PS. The HSQC experiment was also acquired at a ^1^H frequency of 700 MHz for the two polysaccharides with 938 × 1024 data points in *F*_2_ and *F*_1_, respectively, covering 100 ppm in *F*_1_ with uniform and non-uniform sampling of 25 percent sparsity and an exponential weighting set to 100 ms for the *T*_2_ relaxation time. The ^1^H,^13^C-HSQC experiment on the O125ac PS with aliasing instead used 1270 × 1024 data points in *F*_2_ and *F*_1_, respectively, with a spectral width in *F*_1_ of 30 ppm centered at 65 ppm.

### Bioinformatics analysis

The bioinformatics analysis was based on published genomes of *E. coli* O125 strains related to O-antigen synthesis and expression (Iguchi et al. 2015; DebRoy et al. 2016; Liu et al. 2020), as deposited at NCBI (GenBank AB812053.1) by Iguchi et al. (Iguchi et al. 2015) and (GenBank KP835694 for O125ab and KP835695 for O125ac) by DebRoy et al. (DebRoy et al. 2016) but also relied on BLAST (Camacho et al. 2009) and CAZy (Drula et al. 2022).

## Funding

This work was supported by grants from the Swedish Research Council (2017-03703) and the Knut and Alice Wallenberg Foundation to G.W. K.U. acknowledges support from the Swedish Research Council (2012-03564).

*Conflict of interest statement*: None declared.

## Supplementary Material

Supporting_information_O125_GC-MS_cwac061

Supporting_information_O125_NMR_220724_cwac061
