# Supplementary material for: Structural elucidation of the O-antigen polysaccharide from Escherichia coli O125ac and biosynthetic aspects thereof
Source: Glycobiology. 2022 Sep 10;32(12):1089–100. doi: 10.1093/glycob/cwac061 (PMC9680116; doi:10.1093/glycob/cwac061)
Supplement: Supporting_information_O125_GC-MS_cwac061 [file supporting_information_o125_gc-ms_cwac061.pdf]

Structural elucidation of the O-antigen polysaccharide from *Escherichia coli* O125ac and biosynthetic aspects thereof

Axel Furevi[a], Klas I. Udekwu[b] and Göran Widmalm[a]

[a] Department of Organic Chemistry, Arrhenius Laboratory, Stockholm University, SE-106 91 Stockholm, Sweden

[b] Department of Aquatic Sciences and Assessment, Swedish University of Agriculture, P.O. Box 7050, SE-750 07 Uppsala, Sweden

Supporting information

Table of contents

Sugar analysis by GC-MS of *E. coli* O125ab and O125ac O-antigens……………………..…page S2

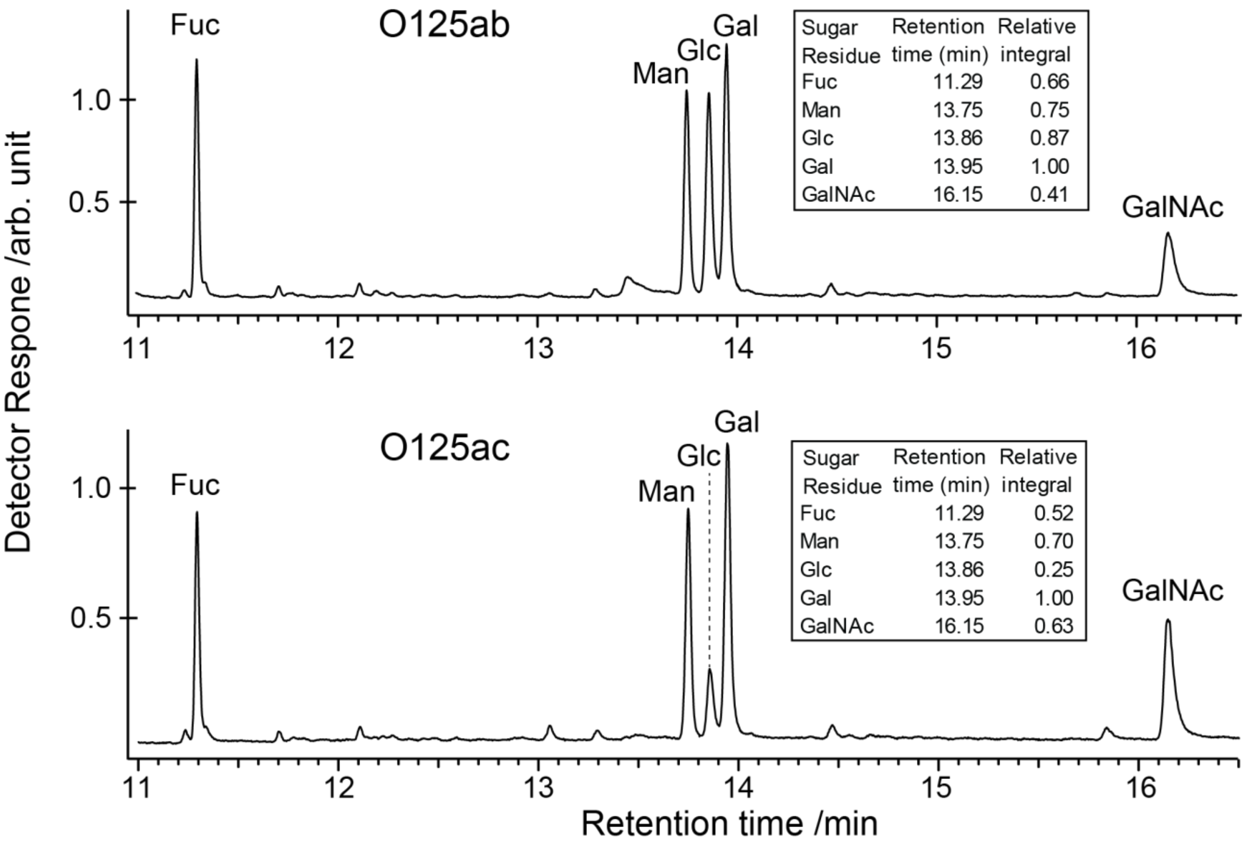

Figure S1. Sugar analysis by GC-MS of alditol acetate derivatives of O-antigen polysaccharides from *E. coli* O125ab (top) and O125ac (bottom). The peaks are annotated with the corresponding sugar residue and their relative integrals are presented in the inserted tables.
